# Tests on Material Compatibility of Phase Change Materials and Selected Plastics

**DOI:** 10.3390/molecules24071398

**Published:** 2019-04-10

**Authors:** Milan Ostrý, Sylva Bantová, Karel Struhala

**Affiliations:** Faculty of Civil Engineering, Brno University of Technology; 602 00 Brno, Czech Republic; bantova.s@fce.vutbr.cz (S.B.); struhala.k@fce.vutbr.cz (K.S.)

**Keywords:** encapsulation, phase change materials, PCMs, heat storage, buildings

## Abstract

Practical applications of Phase Change Materials (PCMs) often require their encapsulation in other materials, such as metals or plastics. This raises the issue of compatibility between PCMs and encapsulating materials, which has still not been sufficiently addressed. The study presented here follows existing research and provides experimental evaluation of the suitability of selected PCMs for proposed integration in building structures. Two organic PCMs, two inorganic PCMs and three representative plastics (polypropylene (PP-H), high density polyethylene (PE-HD) and polyvinylchloride (PVC-U)) were selected for compatibility tests. Evaluation of the results is based on the mass variations of the plastic samples during the test period. Plastic samples were immersed in PCMs and subjected to periodic heating and cooling (for 16 weeks) in a small environmental chamber simulating real operational conditions. The results show that the organic PCMs have a greater ability to penetrate the PE-HD and PP-H compared with the inorganic PCMs. The penetration of all PCMs was most notable during the first four weeks of the experiment. Later it slowed down significantly. Overall, the mass changes in PE-HD and PP-H samples did not exceed 6.9% when immersed in organic PCMs and 1.8% in inorganic PCMs. PVC-U samples exhibited almost negligible (less than 0.1%) mass variation in all cases.

## 1. Introduction

Thermal energy storage systems can solve the time mismatch between energy generation and energy demand and difference in price between peak and off-peak daily hours. Such issues are typical for applications of renewable energy sources, for example, passive solar gains [1]. The applications of renewable energy sources are essential for the design and effective operation of passive houses [2]. Moreover, thermal energy storage can be employed for transport of heat if the heat source and heat consumer, (e.g., a building) are not directly connected. The approach first mentioned for utilizing renewable energy is represented by active systems integrated in building services. Water is commonly utilized as a medium for thermal energy storage and transport in such systems due to its high storage density; especially when the renewable energy or off-peak electricity is considered. These active systems require utility rooms equipped with sensible heat storage units. The second approach is based on direct utilization of passive solar energy gains for heating (to reduce operating costs). The storage capacity of building structures is a key issue in this case.

Latent Heat Storage (LHS) technology represents an advanced approach to heat storage that can be employed when the amount of heat storage medium is limited. Higher heat storage density is caused by absorption or release of latent heat during melting or solidification of storage medium. Moreover, a sensible heat storage process in a solid or liquid state is certainly utilized too. Phase Change Materials (PCMs) for building applications can be selected from three groups [3]:

- Organic PCMs represented by paraffins and non-paraffins
- Inorganic PCMs describes as salt hydrates or metallics
- Eutectics characterized as a mixture of two or more components, for example, organic-organic, organic-inorganic and inorganic-inorganic eutectics

Due to the change in phase the LHS media must be encapsulated and sealed prior to application in buildings. This could be done in several different ways. PCMs can be fully enclosed within a capsule or container (encapsulated). The size of the capsules varies. There are “macro capsules” larger than 1 mm, “micro capsules” between 1 μm and 1 mm and “nano capsules” smaller than 1 μm [4]. The shape of the capsules varies as well. Most notably the marco encapsulation utilizes wide range of capsule shapes: spheres, tubes, pouches or flat containers [5]. Direct impregnation of porous building material with PCM is also possible. The last option is application of shape stabilized PCMs consisting of PCM and supporting material, typically high-density polyethylene and styrene-butadiene-styrene [6]. The type of encapsulation is influenced by the purpose of installation in building structures or building technical systems. Material of encapsulation and methods of encapsulation itself must meet the requirements on [7]:

- Strength and flexibility
- Corrosion resistance
- Thermal stability in desired temperature range of use
- Protection of the environment from harmful interaction with PCMs
- Sufficient surface for heat transfer
- Structural stability and easy handling
- Availability
- Non toxicity

The main issue connected with the application of LHS in buildings is the incompatibility of PCMs and the storage material (capsules). This issue is crucial for estimates of the durability and service life of LHS components and systems. Unfortunately, the number of published studies dealing with the issue is rather small. Existing studies could be divided based on material of the capsules, commonly metals and plastics.

Metals are good heat conductors. Therefore, their application for PCM encapsulation is rather attractive. For example, Khan et al. [8] evaluated the compatibility of (commercially available) salt-hydrate-based PCMs and metal containers. They concluded that only stainless steel was compatible with (most of) the tested PCMs. Similarly, Ushak et al. [9] described the corrosive effect of salt hydrates (PCMs) on three types of metal (potential container materials). The evaluation was based on the mass loss in time. Copper was the most affected metal in the evaluation, while stainless steel was the least affected. Similar tests evaluating corrosion rates through mass loss of the metal (and plastic) samples in time were previously published by Ferrer et al. [10] or Monero et al. [11]. In fact, the issue of corrosion is so obvious that Krishna et al. proposed an evaluation methodology based on American Society for Testing and Materials (ASTM G1) standards [12]. More complex compatibility evaluation was presented by Sari et Kaygusuz [13]. They evaluated the compatibility of stearic, palmitic, myristic and aluric acids (PCMs) and stainless steel, carbon steel, aluminium and copper (potential capsule materials). Their research is notable especially for application of several analytical methods, such as gravimetric analysis (mass loss in mg cm^−2^), corrosion rate (mg day^−1^), microscopic and metallographic investigation. Browne et al. [14] utilized gravimetric analysis and corrosion rate for evaluation of compatibility of several metals and one plastic (potential capsule materials) with three fatty acids, salt hydrate and Micronal PCM. They recommended stainless steel as the best material for the encapsulation of the tested PCMs. Moreover they concluded that aluminium, copper and brass are suitable for application with fatty acids if some corrosion is acceptable.

Plastics represent a relatively low cost materials for encapsulation compared with metals and metal alloys. Plastics are widely used in the construction sector, for example, polypropylene and polyethylene are common materials in building services (e.g., pipes). This seemingly supports their application for PCM capsules. Unfortunately, plastics have poor thermal conductivity and thus the capsule walls have to be as thin as possible.

Generally, the evaluation of compatibility between plastics and PCMs (or chemical solutions in general) is seldom analysed in literature. Results in existing papers are commonly based on the mass variation [15] or on the volume change [16] of the plastics after exposure time. Polypropylene, high density polyethylene, polyethylene terephthalate and polystyrene as representatives of group of plastics were tested as in combination with selected PCM for proposed cold storage by Oró et al. [17]. Their compatibility evaluation was based on monitoring of mass changes during 12 weeks. An interesting study on compatibility of plastic containers and PCMs was published by Lázaro et al. [18]. They reversed the common procedure of immersing plastics into PCMs. Instead they filled PCMs into bottles made of different plastics. They utilized gravimetric analysis for evaluation of interactions between PCMs and the tested plastics.

The results of compatibility tests are clear. Selection of proper PCM-capsule pairs is crucial for long-term applications in buildings. The consequences of undesirable interaction between PCMs and their capsules were summed, for example, by Vasu et al. [19]:

- Contamination of fluids and perforation in vessels and pipes;
- Reduction of container wall thickness leads to loss of mechanical strength and structural failure of breakdown;
- Mechanical damage to major components and added complexity of equipment;
- Loss of technically important surface properties of component;
- Reduced value of goods due to deterioration of appearance.

**Figure 1:**
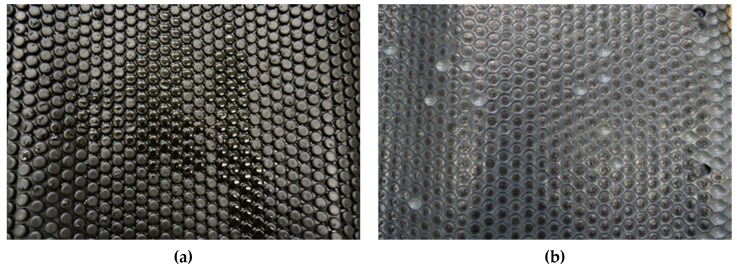
Phase change materials (PCMs) encapsulated in bubble foil: (**a**) View of liquid PCMs on the surface; (**b**) Empty and deformed bubbles with visible solid PCMs on the surface.

Figure 1 illustrates the authors’ own experience with the necessity of PCM-capsule compatibility tests. It shows inorganic PCM encapsulated in a bubble foil. This product can be used as heat storage layer situated above suspended ceiling in office buildings. The layer of plastics is very thin. Serious leakage of PCMs encapsulated in bubbles was observed after few years of use. The same problem was detected in case of plastic tubes and aluminium container panels just after three years of operation. It illustrates the necessity of investigation of compatibility between PCMs and surrounding material. There is no evidence whether inorganic PCMs destroyed the plastic layer by undesirable interaction between PCM and plastic envelope only. Bad joints between bubbles and damage by periodic changes in volume could be also the reason for the leakage. All aspects should be taken into account during the design of any storage component or system. But material compatibility seems to be the most important criterion for assessment of the suitability of the proposed PCM–capsule (plastic) pairs.

The research described in this paper focuses on three kinds of plastics with wide use in building practice. Metals and metal alloys were excluded because the procedure for evaluation of their compatibility with PCMs is different.

## 2. Results

Interaction between samples of three plastics and four PCMs is expressed by change in mass of the samples immersed in each PCM. The experimental procedure and evaluation methodology are described in Section 4. Table 1 shows the monthly mass changes of all tested plastic-PCM combinations. Significant mass change in polypropylene (PP-H) and polyvinylchloride (PVC-U) samples immersed in organic and inorganic PCMs is visible on the first sign. PVC-U samples were almost inert to PCMs in all cases and measured changes were negligible compared to the other plastics tested.

It should be noted that no visual changes were observed on the surface of the samples after testing. Only residual amounts of PCMs were detected on the surface after removal from beakers. This indicates that even long-term exposure in PCMs does not affect the shape of the tested samples. Still, all samples were cleaned and dried before weighing.

**Figure 2:**
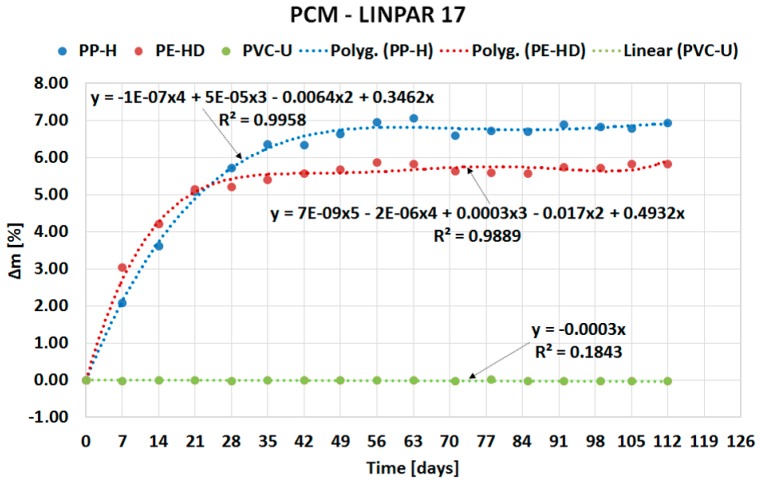
Dependence of mass change of tested plastics on duration of the experiment for Linpar 17.

**Figure 3:**
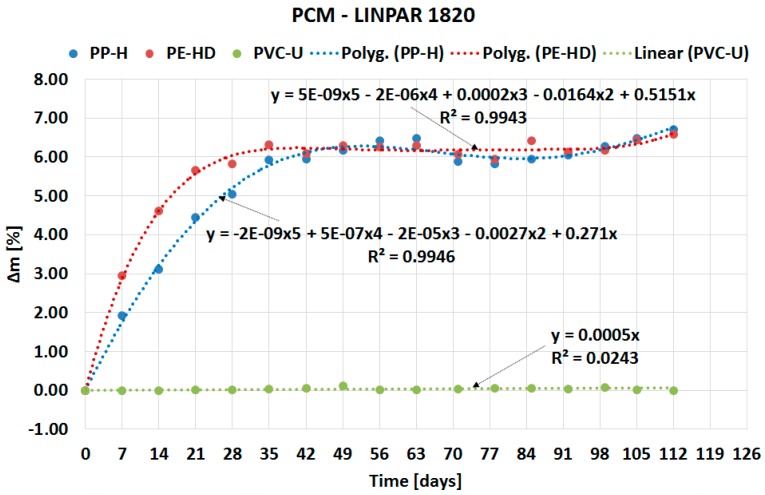
Dependence of mass change of tested plastics on duration of the experiment for Linpar 1820.

**Figure 4:**
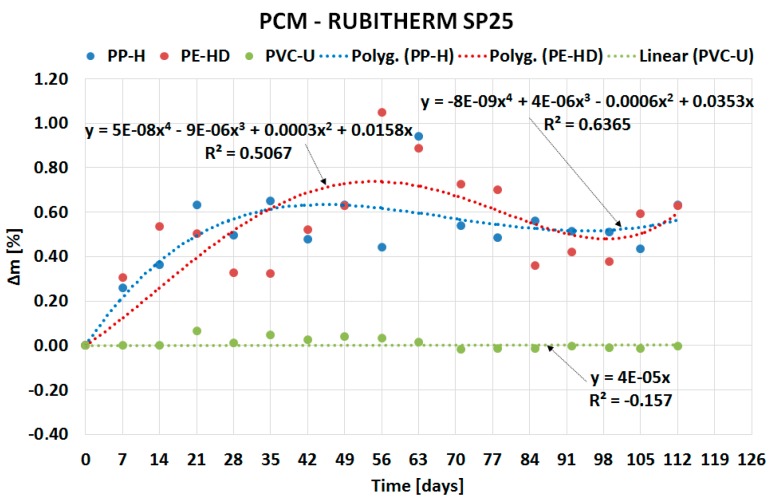
Dependence of mass change of tested plastics on duration of the experiment for SP25.

**Figure 5:**
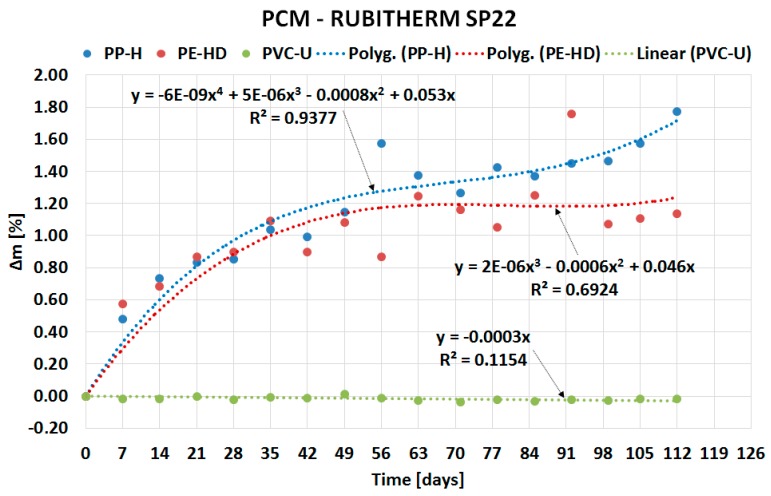
Dependence of mass change of tested plastics on duration of the experiment for SP22.

**Figure 6:**
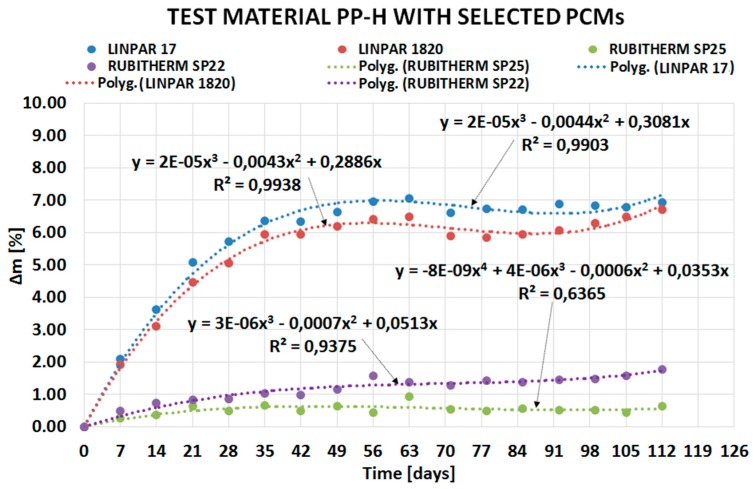
Comparison of the dependence of mass change of polypropylene (PP-H) caused by particular PCMs.

The following sub-sections describe the results of the experiment divided based on the type of PCM: organic (Section 2.1) and inorganic (Section 2.2). The course of PCMs’ penetration into the matrix of the tested plastics is expressed by separate charts in Figure 2, Figure 3, Figure 4 and Figure 5. Overall comparison of the results is provided in Section 2.3 and Figure 6.

### 2.1. Organic PCM results

The curves in Figure 2 demonstrate fast penetration of organic PCM Linpar 17 into high density polyethylene (PE-HD) and PP-H during the first four weeks. Contrary to this behavior, the mass change of PVC-U is negligible. Similar behavior was observed (see Figure 3) in the second organic PCM, Linpar 1820. The mass change during the remaining part of the experiments was almost the same in both PCMs. This means that the tested plastic samples were almost fully saturated by the PCMs during the first four weeks of the experiment. In the end the maximum mass changes did not exceed 7% in both PCMs.

### 2.2. Inorganic PCM Results

The penetration of inorganic PCMs into the matrix of the tested plastics is demonstrated in Figure 4 and Figure 5. PVC does not exhibit any significant mass changes again. The mass change in PE-HD and PP-H is lower compared to organic PCMs. The maximum mass change of 1.8% was achieved with PP-H immersed in Rubitherm SP22.

Figure 4 and Figure 5 show similar behavior of the samples in the first weeks of experiments as Figure 2 and Figure 3: rapid penetration of PCM. However, contrary to organic PCMs, a small mass growth was recorded even during the rest of the experiment.

### 2.3. Overall Results

Figure 6 shows the comparison of mass changes of PP-H in all PCMs. The reason is that this plastic achieved the highest mass increase during the experiment. Therefore, the figure well illustrates the difference in mass change due to the immersion of organic and inorganic PCM. It should be noted that all the values could have been affected by errors in weighing. Polygonal functions calculated for measured data were used for better demonstration of mass change trends during the experiments in the figures.

## 3. Discussion

The results presented in the previous section demonstrate differences in potential compatibility of PP-H, PE-HD and PVC-U with four organic and inorganic PCMs. They show that salt-hydrate-based PCMs have a smaller ability to penetrate the matrix of the tested plastic samples. Moreover, PVC-U was almost without mass change during immersion in all PCMs. Literature provides several studies for comparison of the PP-H and PE-HD results. Sadly, no comparable studies dealing with PVC-U were identified by the authors.

The recorded course of mass changes of PP-H and PE-HD confirm results from experiments with paraffin RT20, RT25 and RT27 described by Castellón et al. in [20]. They observed the same trend of rather fast initial saturation followed by minimal mass changes later for PP, PE-LD and PE-HD. Similarly to the research presented, Castellón et al. also measured lower mass changes of the specified plastics with salt-hydrate-based PCMs. Differences in results could be related to improvements in test procedure (when compared with [20]) applied in the paper presented. These include no contact between samples and complete immersion of the samples by PCMs due to their anchoring in special foam.

Another work dealing with the same issue is [17]. It shows significantly lower material saturation of PP, HDPE, PET and PS compared to the study presented. However, the results are rather incomparable due to the use of different inorganic PCMs. Also, the accuracy of the results in [17] is likely lower as only one sample was evaluated each week and steady-state surrounding conditions were applied. The duration of the experiment in [17] was lower than standard recommendations too.

The experiment with plastic bottles presented in [18] also confirms the differences in mass variation of plastics in contact with organic and inorganic PCMs. However, it describes deformation of plastic samples, which was not observed during the work presented.

## 4. Materials and Methods

**Figure 7:**
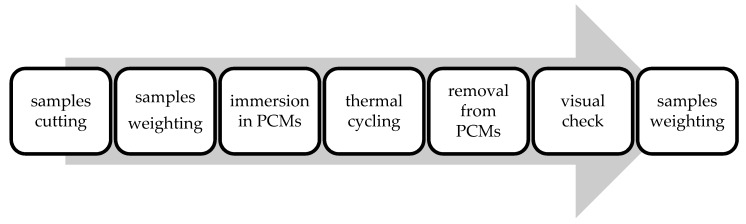
Individual steps of the experimental procedure.

The experiment described verifies the compatibility of selected plastics and PCMs. The aim of the experiment is selection of the most suitable material pairs for future development of PCM applications. The experiment applies gravimetric analysis to quantify the rate of infiltration of PCMs into the plastic samples. The experimental procedure is presented in Figure 7. It was selected based on literature review. Kass et al. [21] evaluated 18 plastics in contact with neat bio-oil using a similar modified procedure. Other works utilizing a similar approach include for example [14,15].

Four PCMs were selected for testing as representatives of LHS media applicable for improving of heat storage capacity of buildings: salt-hydrate-based Rubitherm SP22 and Rubitherm SP25 and paraffin-based Linpar 17 and Linpar 1820. All of them are commonly available on the global market. Table 2 shows parameters of these PCMs obtained with Different Scanning Calorimeter (DSC) measurement. The amount of latent heat varies between 122 and 152 J·g^−1^ and the peak temperature between 22 and 28 °C.

Three plastics were selected as potential materials for PCM capsules and tested: PP-H (polypropylene), PE-HD (high density polyethylene) and PVC-U (polyvinylchloride). Main parameters of selected plastics are presented in Table 3. Generally, these plastics were selected based on their availability on the market, low costs and the fact that they are commonly utilized in the packaging industry. PP-H represents the group of polyolefins and is classified as semi crystalline thermoplastic polymer. It is flammable material with low thermal conductivity currently used, for example, in sewerage or drinking water supply systems. PE-HD is a thermoplastic polymer as well. Its applications in the building industry include rainwater pipes or storage tanks, and so on. PVC-U represents vinyl group of polymers. It is a hardened variant of common PVC. PVC is widely used in the building industry. Its applications include waterproofing sheets, window frames or flooring.

**Figure 8:**
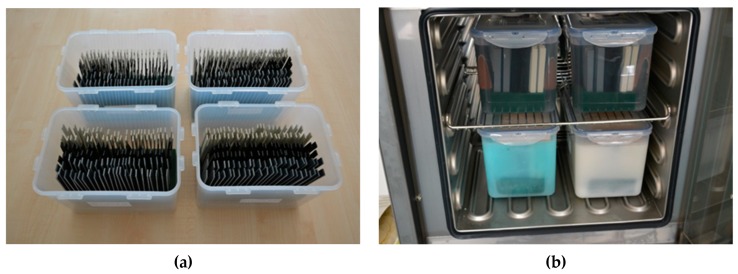
Plastic samples in the test beakers: (**a**) Samples before immersion in PCM and thermal cycling; (**b**) Samples immersed in PCMs placed in small environmental chamber during testing.

The gravimetric method defined in international standard ISO 175:2000 [22] was utilized to set the initial conditions and subsequently perform the experiment. Considered conditions of the experiment include:

16 weeks immersion (long-term test) of plastic samples in beakers with PCMs (see Figure 8b).

Repetitive four-stage temperature cycles in an environmental chamber during immersion. 

Individual four-hour stages were: increasing the temperature to a maximum of 40 °C; maintaining the temperature at this level; decreasing the temperature to 15 °C and maintaining the temperature at this level too. This temperature interval was applied due to the need for complete solidification or melting of PCMs in the beakers: the temperatures were set approx. 10 °C bellow and 10 °C above peak temperatures of the tested PCMs.

64 plastic sample sets (16 sets per one PCM; see Figure 8a). Each sample set consisted of three individual plastic plates (100 × 10 × 1.5 mm) to minimize the impact of possible defective samples on the results.

Sample sets were gradually removed from the PCMs in seven day periods for measurements.

Figure 8a shows the beakers with all the plastic samples (without PCMs) prior to testing. The samples were cleaned, visually inspected (for impurities, damage, etc.) and marked beforehand. The figure also shows mutual separation of individual samples in the beakers. This ensured even exposure of the whole surface of the samples to the PCM—a parameter that was not fully addressed in the reviewed works, such as [12]. Figure 8b shows the start of the 16-week testing in the environmental chamber. Beakers with colorless organic PCMs are on top and beakers with blue and white inorganic PCMs are at the bottom of the chamber.

The evaluation procedure described in [22] is based on monitoring of the mass changes in the tested plastic samples. Therefore, the sample sets were gradually removed from the beakers with PCM to perform measurements of their mass (after throughout water cleaning and visual check). It should be highlighted that the measurements were performed immediately after the removal from the beakers and cleaning to avoid degradation and fall-off of the PCM (e.g., evaporation) on the samples. The percentage change in mass was calculated for every sample using Equation (1). Analytical Balance 220 g × 0.1mg was used for samples weighting. The measured mass of each sample was rounded to 0.1 mg (based on the accuracy of the balance). Then the median value for each sample set was calculated and the sample corresponding with this value was included in subsequent interpretation (see Section 3). The median method was utilized to separate any extreme results caused by defective samples.

∆m = [(m_2_ − m_1_)/m_1_] × 100 (1)

where Δm is the percentage change in mass in %, m_2_ is weight of the sample (in g) after removal from PCMs and m_1_ is initial weight of the sample (in mg) before immersion in PCM

## 5. Conclusions

The paper presents evidence of interaction between PCMs and plastics potentially usable for the PCM encasement. The experiment evaluating compatibility of three plastics with two organic and two inorganic PCMs was performed for this purpose. The experiment was based on the gravimetric method and evaluated the potential of PCMs’ migration into the plastic samples. The results of the experiment (see Figure 2, Figure 3, Figure 4, Figure 5 and Figure 6) show that the mass of the samples increases depending on the length of immersion in PCMs. Most notable mass changes occurred during the first four weeks after immersion. This indicates long-term stability of plastics after the initial saturation. Overall, the mass changes of PVC-U samples in contact with all PCMs were almost negligible (less than 0.1%). The highest mass change (up to 6.9% increase) occurred in PP-H and PE-HD samples immersed in organic PCMs. A lower mass change (up to 1.8% increase) occurred in PP-H and PE-HD samples immersed in inorganic PCMs. Therefore, it could be concluded that PVC seems to be the best material for (tested) PCM encapsulation. However, it should be noted that the contemporary construction sector is abandoning PVC applications (in the Czech Republic).

Undesired interactions between PCMs and their capsules could significantly reduce service life of the LHS systems (in buildings). Existing literature (e.g., [20]) indicates that penetration of PCM could affect the characteristics (e.g., modulus of elasticity) of the encapsulating materials. Any change of structural characteristics in turn affects the ability of the capsules to withstand periodical volume changes of the PCMs. Especially the joints are prone to mechanical failures. Therefore, the results of the presented experiment will be utilized during following investigation of the characteristics of plastics during long-time exposure to PCMs. Such investigation should provide data for development of stable and efficient LHS applications in buildings.

## Figures and Tables

**Table 1:** Percentage change in mass of tested plastics depending on used PCMs through time.

Material (Plastics)	Time	Percentage Change in Mass Δm [%]			
		Linpar 17	Linpar 1820	SP25	SP22
PP-H	4 weeks	5.7175	5.0474	0.4968	0.8547
	8 weeks	6.9493	6.4214	0.4414	1.5721
	12 weeks	6.6993	5.9488	0.5609	1.3682
	16 weeks	6.9399	6.7153	0.6326	1.7722
PE-HD	4 weeks	5.2100	5.8225	0.3294	0.8979
	8 weeks	5.8614	6.2467	1.0484	0.8704
	12 weeks	5.5740	6.4250	0.3588	1.2533
	16 weeks	5.8169	6.5783	0.6281	1.1381
PVC-U	4 weeks	−0.0285	0.0201	0.0107	−0.0199
	8 weeks	−0.0137	0.0244	0.0333	−0.0106
	12 weeks	−0.0310	0.0624	−0.0141	−0.0312
	16 weeks	−0.0256	0.0000	−0.0031	−0.0172

**Table 2:** Parameters of selected PCMs (measured during melting).

Type	Product Name	Manufacturer	Latent Heat [J∙g^−1^]	Onset Temperature [°C]	Peak Temperature [°C]
inorganic	SP22	Rubitherm	145	14	25
inorganic	SP25	Rubitherm	122	18	28
organic	Linpar 17	Sasol	152	21	22
organic	Linpar 1820	Sasol	141	24	27

**Table 3:** Parameters of selected plastics.

Material	Thermal Conductivity [W/(m·K)]	Modulus of Elasticity [MPa]	Density [g/cm^3^]	Melting Point [°C]
PP-H	0.22	1350	0.92	90
PVC-U	0.20	3000	1.43	80
PE-HD	0.43	1000	0.95	75
